# Macro-meso damage characteristics of coal body under different pressure relief conditions

**DOI:** 10.1038/s41598-024-55795-y

**Published:** 2024-03-11

**Authors:** Jinyu Li, Jiawei Pang, Lei Wang, Hongbao Zhao, Yilong Zhang, Tao Wang

**Affiliations:** 1 College of safety science and engineering, Liaoning Technical University, Huludao, 125105 Liaoning China; 2 Key Laboratory of Mine Thermodynamic disasters and Control of Ministry of Education (Liaoning Technical University), Huludao, 125105 Liaoning China; 3 State Key Laboratory of Mining Response and Disaster Prevention and Control in Deep Coal Mines, Anhui University of Science and Technology, Huainan, 232001 Anhui China; 4 School of Safety and Emergency Management Engineering, Taiyuan University of Technology, Taiyuan, 030024 Shanxi China

**Keywords:** Pressure relief path, Activation damage, Particle flow theory, Acoustic emission, Applied physics, Mineralogy

## Abstract

The mining of the protective coal seam usually produces different pressure relief effects on the different areas of protected coal seam, the reason is that the stress paths of protected seam coal body in different areas caused by mining effect are different. In order to explore the differential pressure relief damage effect of coal body under different pressure relief conditions, the stress evolution path of coal body in different areas of the protected coal seam is obtianed by using theoretical analysis and the macro–micro damage characteristics of coal body under different stress paths by using numerical simulation in this paper. The results show that: The damage characteristics of the sample models are basically the same in the in-situ stress recovery stage and the mining disturbance stage of the two stress paths. With ith the sequence of stress stages experienced by the sample model, the distribution of acoustic emission events concentrates in the high-intensity area and the porosity continues to decrease. The number of cracks increases slowly in the stage of in-situ stress recovery stage, most of which are tensile cracks, while the number of cracks increases sharply in the mining disturbance stage, most of which are shear cracks. The difference of the deformation and macro meso damage characteristics of the sample models under the two stress paths is mainly reflected in the post mining pressure relief stage. At the post mining pressure relief stage of path 1, the number of cracks in the sample has little growth, and most of them are small energy tensile cracks, and the porosity increases, which verifies its obvious pressure relief activation antireflection effect; At this stage of path 2, the crack growth of the sample is obvious, and most of them are high-energy shear cracks, and the porosity continues to decrease. Compared with path 1, the pressure relief expansion effect of the sample model is suppressed and the compression damage continues to develop in this stage of path 2.

## Introduction

With the normalization of "deep" mining of coal resources, the high gas and low permeability coal seams are more and more frequent occurrence (2019), pressure relief of protective coal seam mining assisted by gas drilling are widely used as an effective processing technology to solve the above problem. However, due to the different spacing, thickness and mining technology of coal seam, the pressure relief path of the protected coal seam will be different, resulting in different pressure relief effects. At the same time, the degree of pressure relief and the range of high-efficiency pressure relief area of protected coal seam are the key parameters to guide the layout of gas drilling boreholes. Therefore, it is particularly important to find out the evolution characteristics of coal damage and gas migration channel under different pressure relief conditions.

Experts in the field have conducted a lot of research on the damage characteristics of coal and rock mass under different pressure relief paths, mainly focusing on the deformation, damage and permeability characteristics of coal and rock mass. Gong et al.^1^ obtained the damage degree and evolution law of permeability of coal and rock mass under mining disturbance conditions by combining experiments and theoretical analysis; Zhang et al.^2^ obtained the mechanical and thermally damage characteristics of coal and rock mass based on the triaxial cyclic loading and unloading test, and revealed the temperature-peripheral pressure effects on rock damage properties in the process; Yang et al.^3^ studies the mechanical and energy damage evolution law of coal and rock mass under cyclic loading and unloading, and obtained the coupled evolution equation of coal and rock damage dissipation energy with cyclic loading times and confining pressure.; Xia et al.^4^ obtained the mechanical and crack damage evolution characteristics of sandstone under the stress disturbance of fully mechanized caving mining through similar simulation experiments; Gao et al.^5^ defined the structural damage variable of coal and rock mass based on the damage and failure characteristics of coal and rock mass, and gave the change rule of the damage degree and permeability coefficient of the protected coal seam; Li et al.^6^ studied the dynamic crack growth law of coal samples during loading and unloading by PFC softword based on the fracture mechanics and deformation characteristics studied by experimental study. Yu et al.^7^ used PFC numerical simulation to obtain the causes of crack initiation and propagation of prefabricated fractured rock samples under different loading and unloading rates, which effectively supported the conclusions of indoor physical tests; Gao et al.^8^ used PFC numerical software to carry out numerical simulation tests of equal amplitude periodic load at three different stress levels, and analyzed the influence of periodic load stress levels on the failure modes, acoustic emission counting and damage evolution characteristics of coal samples.

In summary, many previous scholars have systematically studied the damage characteristics of coal and rock mass under different stress paths, and have achieved fruitful results. It is generally accepted in the field that experimental research is the most accurate research method to obtain the stress damage development of coal and rock mass, and the obtained laws are also the most authentic and scientific. However, due to the shortage of test equipment, the damage evolution of coal and rock samples during loading can only be indirectly reflected by specific parameters. Even if individual high-precision equipment can realize the visualization of damage process, it is rarely reported due to high cost and low popularity. Therefore, numerical simulation has become a good auxiliary means to study the damage evolution characteristics of coal and rock because of its advantages of realizing the damage visualization of the specimen in the whole process of loading. As far as the PFC software used in this paper is concerned, it has been successfully applied to the study of coal and rock mechanics, damage and fluid solid coupling characteristics (Wang et al.^9^, Mu et al.^10^, Wu et al.^11^), and compared with the conventional PFC numerical simulation, this paper has realized the following innovation: ① The complex loading and unloading of specific stress path in PFC numerical simulation is realized; ② the acoustic emission monitoring program for the damage of the sample model at the meso scale is established by software secondary development based on moment tensor theory. Therefore, the macro and micro damage characteristics of coal body damage under mining effect are systematically studied.

## Research scheme

### Engineering background and stress analysis

During the exploitation of the protective coal seam in the deep environment, the roof strata collapse and deformation induced by the mining will not involve the surface rock soil layer, Therefore, the bearing pressure of the floor formed by the mining effect will be less than the original self-weight stress, Therefore, it will have the effect of pressure relief activation and antireflection on its protected coal seam.

**Figure 1:**
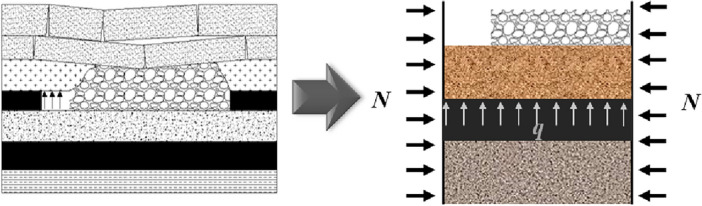
Load characteristics of floor in upper protective layer mining (Qian et al.^12–14^).

**Figure 2:**
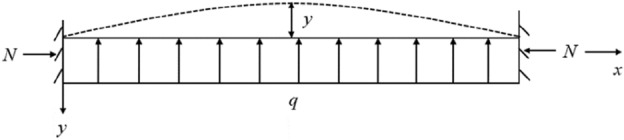
Stress model of protected coal seam (Qian et al.^12–14^).

Since the thickness of the coal seam is far less than the length in the horizontal direction, based on the plate structure characteristics of the key layer and the boundary conditions of the unloading space of the goaf, the structural model of the goaf floor of the working face can be simplified as a rectangular thin plate with fixed supports under uniform load and lateral pressure without the influence of faults, as shown in Fig. 1. By further simplifying it, the stress state of the protected coal seam can be transformed into the stress model shown in Fig. 2.

Define the deflection deformation at the midpoint of the mechanical model shown in Fig. 2 as *y*, and the moment at any point is in equilibrium, as shown in formula (1):

1 $$\frac{ql}{2}x + Ny - \frac{q}{2}x^{2} = - EI\frac{{d^{2} y}}{{dx^{2} }},$$

where *N* is the horizontal force of the model, and *l* is the length of the model; *q* is the uniform load at the bottom of the model; *E* is the elastic model, and *I* is moment inertia of the cross-sectional of model. Define formula (2) as shown:

2 $$\alpha^{2} = \frac{N}{EI}.$$

Substituting formula (2) into formula (1) and solving the partial differential equation, we can get:

3 $$\begin{gathered} y = \frac{q}{{N\alpha^{2} }}\left( {\tan \frac{\alpha l}{2}\sin \alpha x + \cos \alpha x - 1} \right) + \frac{q}{2N}x^{2} - \frac{ql}{{2N}}x \hfill \\ = \frac{q}{{N\alpha^{2} }}\left( {\sec \frac{\alpha l}{2}\cos \left( {\frac{\alpha l}{2} - \alpha x} \right) - 1} \right) + \frac{q}{2N}x^{2} - \frac{ql}{{2N}}x \hfill \\ \end{gathered}$$

Under normal conditions, the deflection at the midpoint of the model is the largest, that is:

4 $$y = \frac{q}{{N\alpha^{2} }}\left( {\sec \frac{\alpha l}{2} - 1} \right) - \frac{{ql^{2} }}{8N}.$$

The bending moment of model midpoint *M*_*mid*_:

5 $$M_{mid} = \frac{{ql^{2} }}{8} + Ny.$$

Stress distribution on the middle section of the model:

6 $$\sigma = \sigma_{N} + \sigma_{M} = \frac{N}{A} + \frac{{M_{(x)} y}}{I},$$

where *A* is the cross-sectional area of the model; *h* is the height of the model; *I* is the hydrostatic pressure on the model; σ is the stress generated by bending moment.

By taking formula (5) and (6) into formula (4), we can obtain:

7 $$\sigma_{\min } = \frac{N}{A} - \frac{Eqh}{{2N}}\left( {\sec \frac{ql}{2} - 1} \right),\,\,\,\sigma_{\max } = \frac{N}{A} + \frac{Eqh}{{2N}}\left( {\sec \frac{ql}{2} - 1} \right).$$

From the analysis result of formula (7), the stress state of the protected coal body at different positions in the vertical direction is different, The load stress of the upper part of the protected coal seam is less than that of the original self-weight stress, which is in a state of expansion deformation; The stress of the lower part is greater than that of the original self-weight stress, which is in compression state^12,14^.

### Research scheme

**Figure 3:**
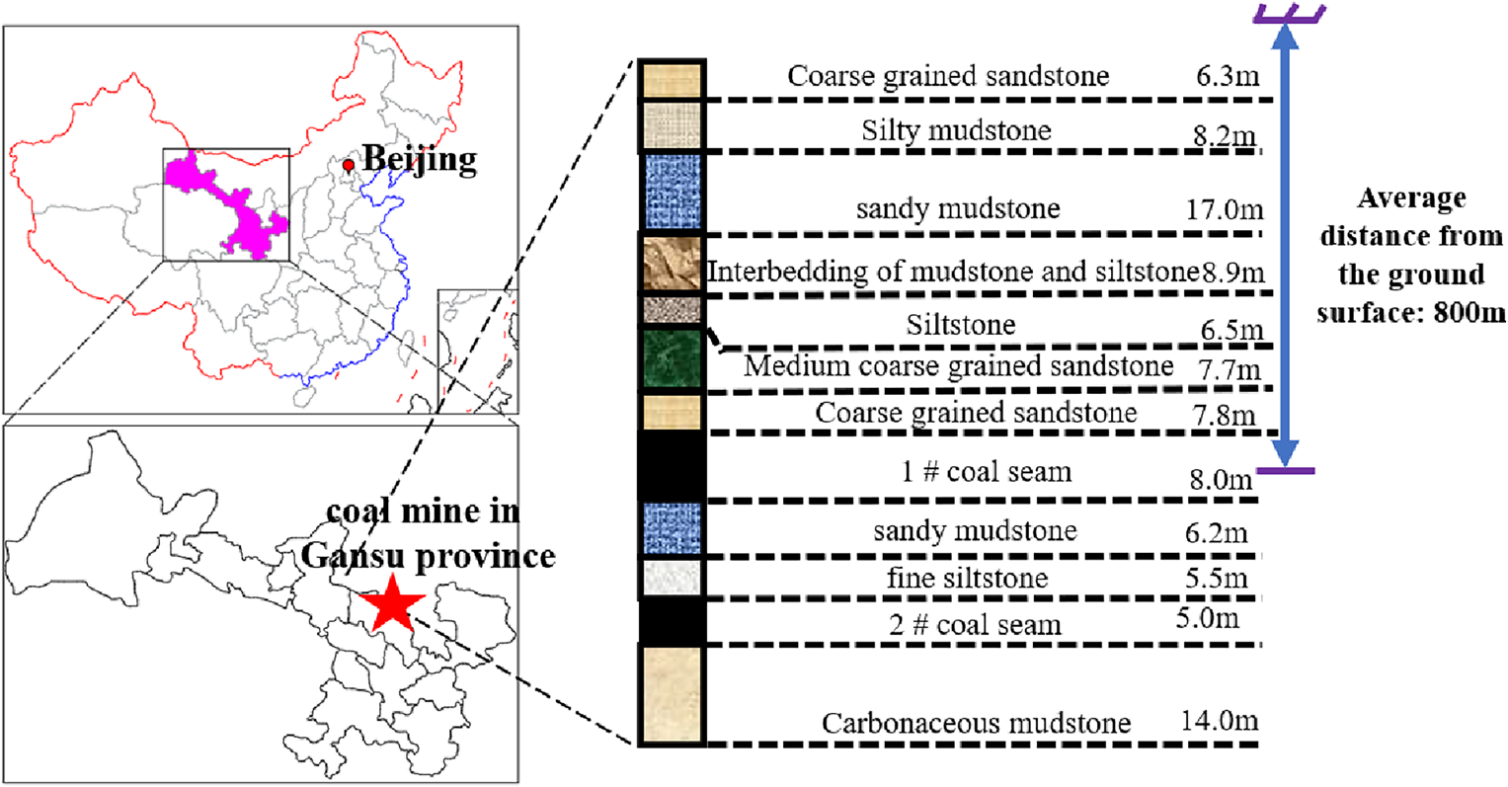
Engineering background geological data.

**Figure 4:**
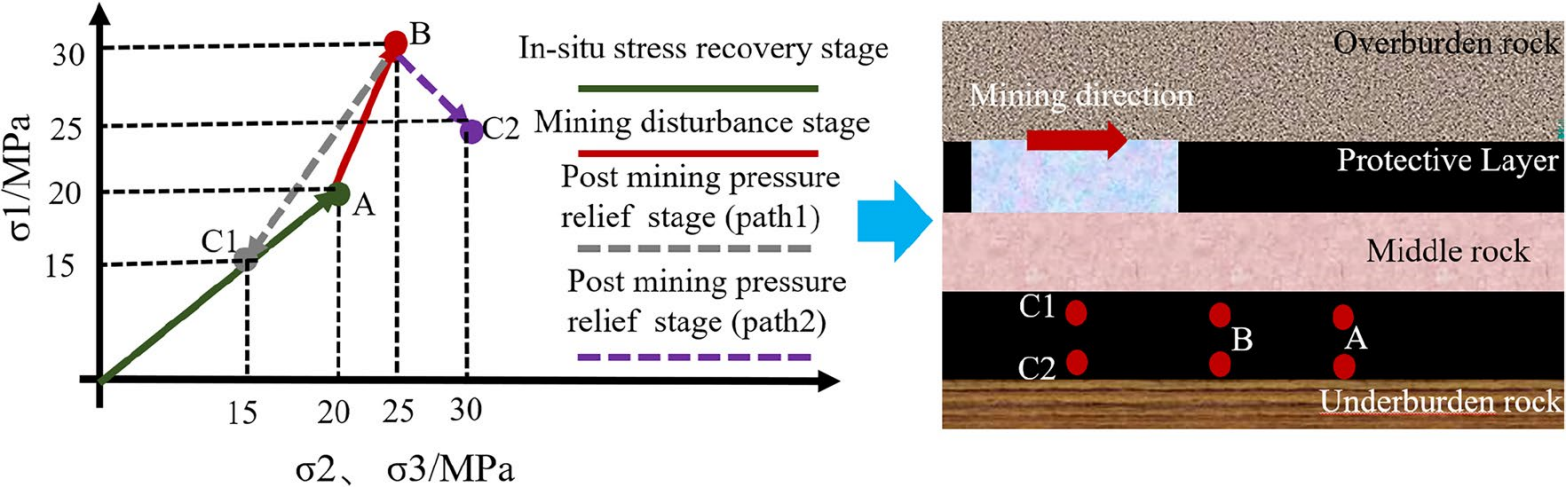
Indoor similar simulated stress path.

Taking the geological conditions of a mine in Gansu Province as the background, as shown in Fig. 3, the average depth of 1 # coal seam of the mine is 800 m. The protected coal seam working face (No. north 1103) has a dip length of 190 m and a strike length of 870 m, at present, the mining of the working face is square. The elastic modulus of coal and rock mass and other basic mechanical parameters are measured by uniaxial compression test. As shown below, the stress concentration factor is taken as 1.5 (Qian et al.^12–14^). The stress evolution path of the coal seam in the protected coal seam under the mining disturbance of the upper protective coal seam is calculated and proposed. Section OA is in-situ stress recovery stage, and point A is in hydrostatic pressure point; Section AB is the mining disturbance stage, and point B is the stress peak. Section BC is the post mining pressure relief stage, BC1 section is unloaded to a stress state less than the original rock stress, as shown in path 1 in Fig. 4; BC2 section is unloaded to a stress state greater than the original rock stress, as shown in path 2 in Fig. 4.

### Model establishment and parameter calibration

**Figure 5:**
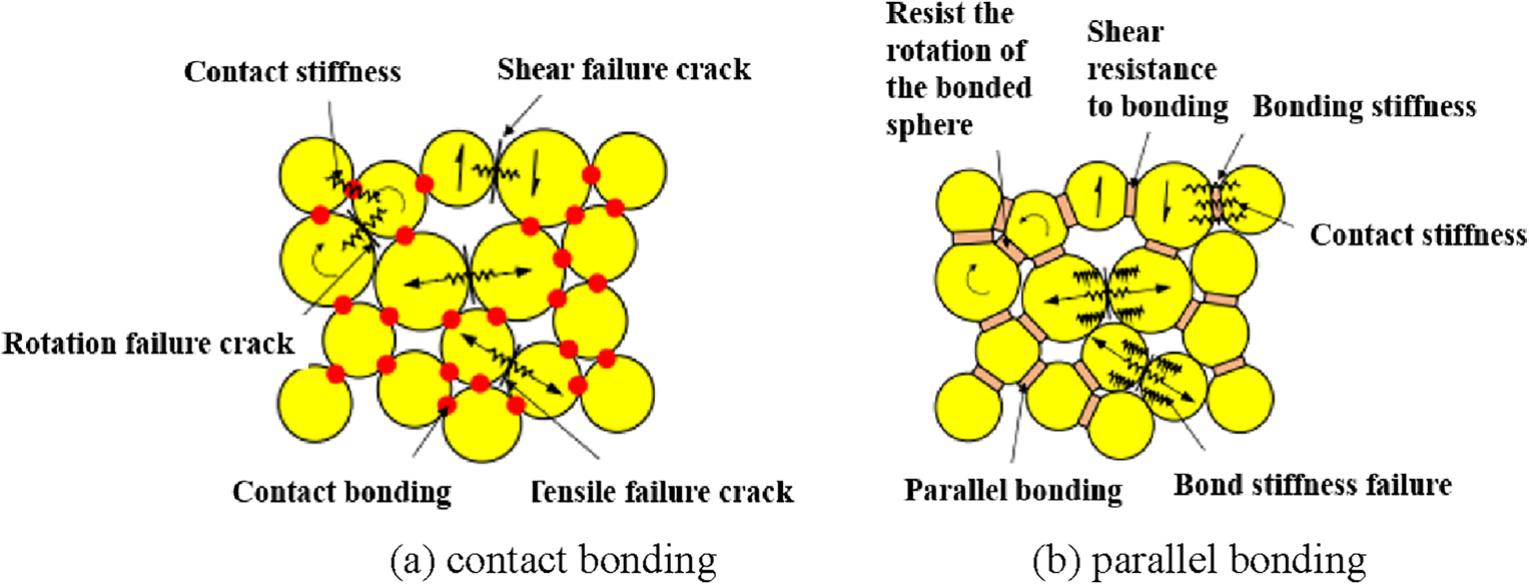
Schematic diagram of PFC particle bonding model.

In the paper, the bonded particle model (BPM) is used to research the macro–micro damage characteristics of coal body under different pressure relief stress paths, the model is specially used to simulate the meso damage process of rock materials in particle flow program (PFC) (Wu et al.^11^). In the process of PFC calculation, there are two bonding modes between model particles: contact bonding model and parallel bonding model, as shown in Fig. 5. The parallel bonding model is similar to the cement like substance connecting adjacent particles, just like a beam, which resists the moment caused by the rotation or shear of particles, the parallel bonding model includes two parts: the contact stiffness and the bonding stiffness. When the bonding breaks, the bonding stiffness is removed, and as long as the particles remain in contact, the contact stiffness still plays a role. Therefore, the parallel bonding model is more suitable for simulating the damage and failure process of coal.

**Figure 6:**
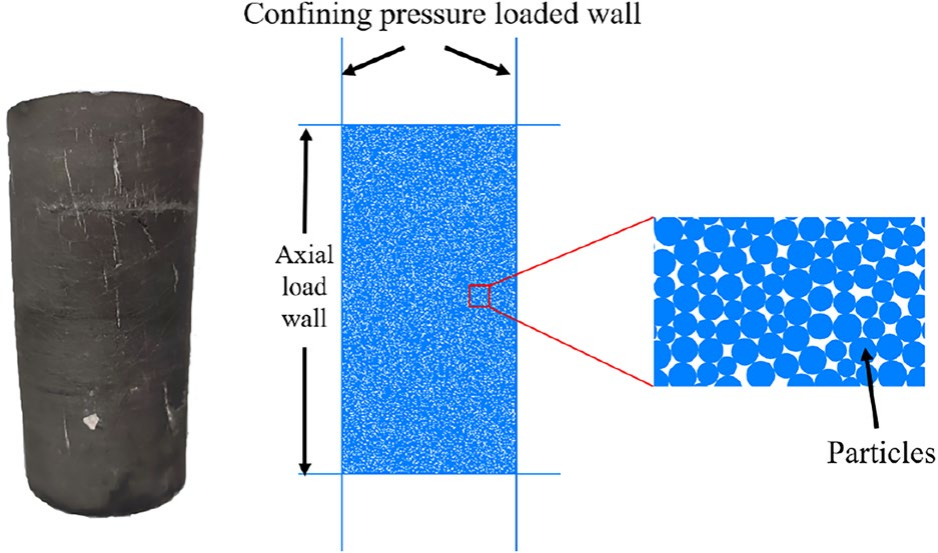
Raw coal sample and sample model.

In order to ensure the rigorism and consistency of the research, The numerical simulation sample model in this paper is set to the same size as the standard raw coal sample used in the indoor test, as shown in Fig. 6, the specific size is 100 mm × 50 mm, the size of the loaded wall should be larger than the size of the sample model, so it is set to 150 mm × 75 mm, finally, in order to ensure that all the above research objects are located in the calculation area, the calculation area is set at 240 mm × 100 mm. In the area surrounded by the axial pressure and confining pressure loading wall, the random seed 10,001 is used to generate round particles to make the sample model, and the final number of generated particles is 12,070.

In the parallel bond model, many meso parameters such as contact modulus, stiffness ratio and bond strength determine the mechanical and damage characteristics of coal and rock mass, the determination of these meso parameters is a prerequisite for obtaining accurate model. At present, the most commonly used method by scholars in the same field is the "trial and error method" recommended by the PFC manual (Wu et al.^11^), that is, constantly adjusting the values of the meso parameters of the sample model until the obtained macro mechanical parameters of the sample model match the indoor test results.

**Figure 7:**
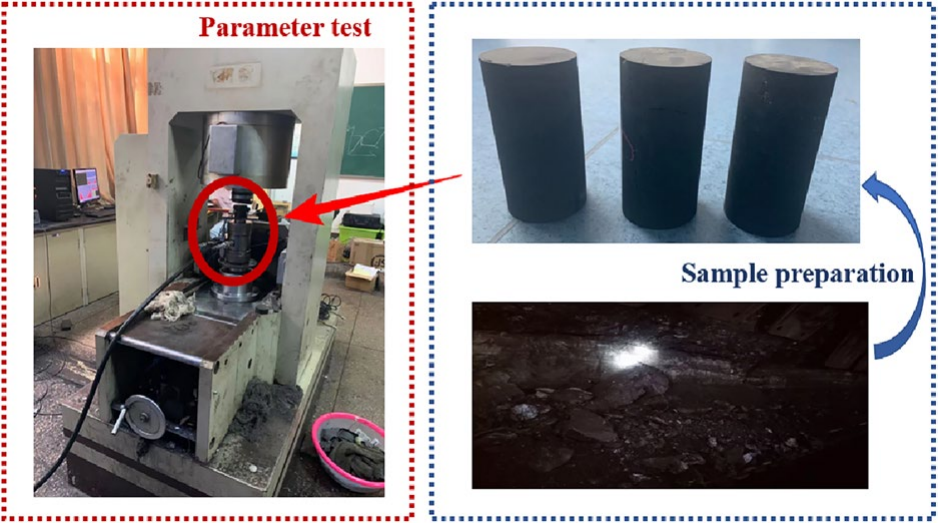
Sample preparation and parameter test.

Therefore, in this paper, the coal blocks with large block size are obtained from the coal wall of the 2 # coal seam engineering background mine, and standard raw coal samples are prepared by coring and grinding. The RMT-150B electro-hydraulic servo is used as the test equipment to carry out uniaxial compression test to obtain its basic mechanical parameters, as shown in Fig. 7.

**Figure 8:**
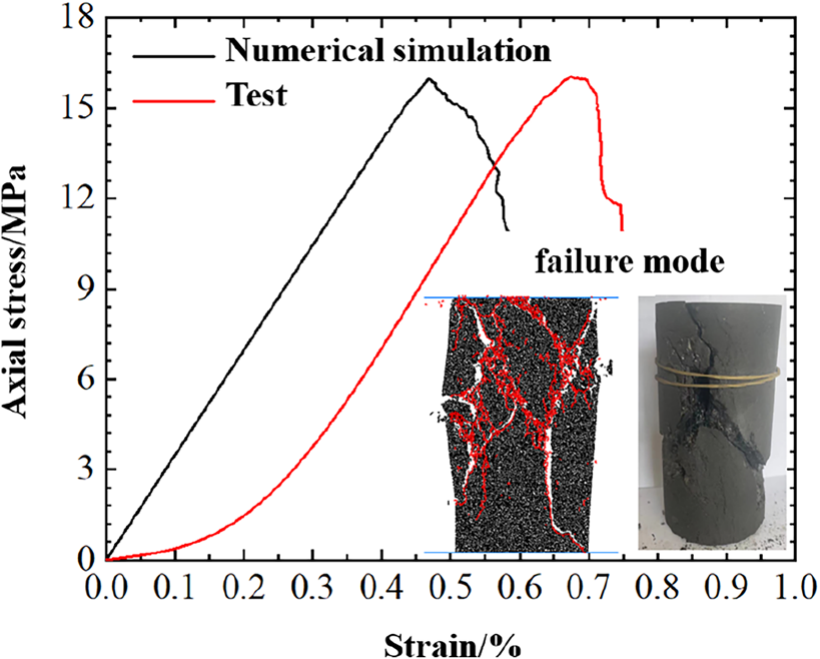
Comparison between indoor test and numerical simulation results.

The displacement loading method was used in the indoor test and the loading speed was set at 0.1 mm/min, Three raw coal samples were tested in parallel in the indoor test, the poisson's ratio, peak stress and elastic modulus of each sample are similar, so the mechanical parameters of one sample are taken as an example to calibrate the parameters. By extracting and comparing the stress–strain curves and post peak failure patterns of raw coal sample and sample model under uniaxial compression, the rationality of selected meso parameters of the sample model is verified, as shown in Fig. 8, the specific basic parameters of particles are shown in Table 1.

**Table 1:** Meso parameters of sample model.

Meso parameters	Value
Minimum particle radius, *R*_min_(mm)	0.25
Particle size ratio, *R*_rat_ = R_max_/R_min_	1.66
frictional coefficient, *μ*	0.3
Particle density, *ρ*(kg/m^3^)	2700
Particle stiffness ratio (normal stiffness / tangential stiffness), *k*_n_/*k*_s_	2.82
Particle contact modulus, *E*_c_(GPa)	1.5
Parallel bonding stiffness ratio (normal stiffness/tangential stiffness), *k*_n_/*k*_s_	2.82
Parallel bonding contact modulus, *E*_c_(GPa)	1.5
Parallel bonding cohesion, *pb_coh*(MPa)	33
Parallel bonding cohesion, standard deviation (MPa)	2
Parallel bonding tensile strength, *pb_ten* (MPa)	27
Parallel bond tensile strength, standard deviation (MPa)	2

It can be seen from Fig. 8 and Table 2, The uniaxial compressive strength of the raw coal sample and the sample model are 15.9 MPa and 16.4 MPa, the elastic moduli are 2.15 GPa and 2.03 GPa, respectively. The deviations of the compressive strength and elastic modulus obtained by the two research methods are 2.6% and 5.6% respectively, which are within a reasonable error range,

**Table 2:** Deviation analysis of experiments and numerical simulations.

	Uniaxial compressive strength/MPa	Elastic moduli/GPa	Deviation/%
Raw coal sample	15.9	2.15	2.6
Sample model	16.4	2.03	5.6

The only difference of the stress–strain curves obtained by the two research methods is that the stress–strain curve of the raw coal sample obtained by the indoor test has an obvious compaction stage, While the stress–strain curve of the sample model in the numerical simulation directly enters the elastic stage, this is due to the internal difference between the raw coal sample and the sample model. The primary fracture structure in the raw coal sample is developed, however, the sample model is "preloaded" before calculation, so the particles in the model were in close contact, and directly entered the elastic deformation stage after the upper and lower loaded walls began to move and pressurize. From the failure modes of both, it can be seen that the macro failure mode of the sample model is near-x-type shear failure, which is caused by meso tension cracking. The macro failure mode of the raw coal sample is also near-x-type shear failure.

In summary, the mechanical parameters and macro failure modes obtained by numerical simulation are in good agreement with the indoor test results, and the calibrated meso parameters can be used for follow-up numerical simulation studies.

### Principle of acoustic emission monitoring based on moment tensor theory

In order to study the evolutionary characteristics of coal body meso-scale damage and gas transport channels, a calculation method using moment tensor theory proposed by Wu et al.^11^ to establish acoustic emission simulations on the meso-scale was investigated with the principles described below:

① Firstly, the particles originally in contact at both ends of the microfracture are defined as the source particles, and after the breakdown of the adhesion between them (micro-rupture), the contact on the source particles will be deformed due to the movement of the source particles, thus causing a change in the contact force. The area of action of the micro-rupture is that the center of the micro-rupture is the center of the circle, and the radius of action is the maximum source particle diameter. Therefore, the change in contact force for all contacts on the source particle is multiplied by the corresponding arm of force (the distance between the position of the contact point and the center of the microfracture), and the summation operation yields the moment tensor component, and the expression is shown in Formula (8):

8 $$M_{ij} = \sum\limits_{S} {\Delta F_{i} } R_{j} ,$$

where $\Delta F_{i}$ is the $i{\text{ - th}}$ component of the contact force variation, $R_{j}$ is the $j{\text{ - th}}$ component of the distance from the contact point to the center of the microfracture. Where the spatial location of the acoustic emission event is performed according to the determination criteria in Table 3.

**Table 3:** Spatial location criteria of acoustic emission events.

Number of fractures included in the acoustic emission event	Acoustic emission event spatial
= 1	Centering of microfractures
> 1	Geometric center of all microfractures

**Figure 9:**
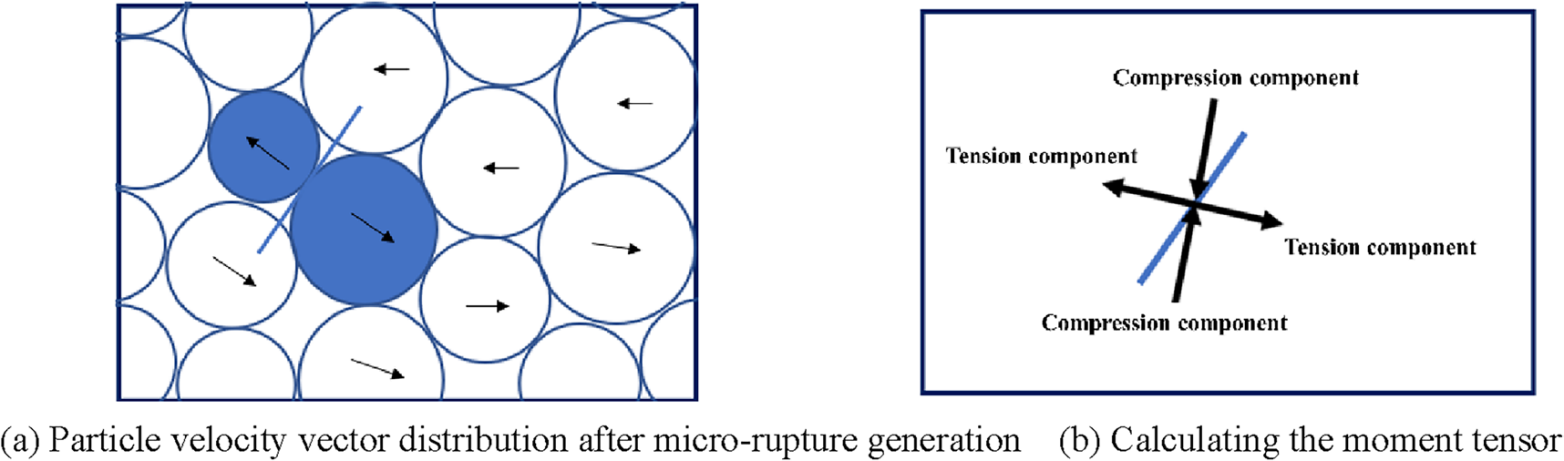
There is only one AE event of tensile fracture in the monitoring range.

As shown in Fig. 9 for only one acoustic emission event within the monitoring range, in Fig. 9a, the source particle velocity vector indicates that the source particle is moving rapidly to both sides perpendicular to the microfracture after the microfracture is generated. In Fig. 9b, the moment tensor is calculated by Formula (8), and the length and direction of the 2 sets of arrows are calculated and represented by the eigenvalues of the moment tensor matrix. Through the moment tensor as shown in Fig. 9b, it can be found that after the microfracture is created, the microfracture opening expands perpendicular to the microfracture to both sides, and there is a compressive component of the moment tensor pointing to the center of the microfracture, indicating that the upper and lower particles move toward the opening area where the microfracture is created under the action of external forces.

② In the calculation process, the moment tensor with the maximum scalar moment value is used as the moment tensor for each firing event and stored to improve the calculation efficiency. According to the moment tensor matrix, the expression of the scalar moment is shown in Formula (9):

9 $$M_{0} = \left( {\frac{{\sum\limits_{j = 1}^{3} {m_{j}^{2} } }}{2}} \right)^{{{\raise0.7ex\hbox{$1$} \!\mathord{\left/ {\vphantom {1 2}}\right.\kern-0pt} \!\lower0.7ex\hbox{$2$}}}} ,$$

where $m_{j}$ is the $j{\text{ - th}}$ eigenvalue of the moment tensor matrix.

③ Based on the peak scalar moment of the acoustic emission event moment tensor, the rupture intensity M of the acoustic emission event can be calculated from Formula (10):

10 $$M = \frac{2}{3}\lg M_{0} - 6.$$

The duration of the acoustic emission event can be calculated assuming that the propagation velocity of the rupture extension is half of the rock shear wave velocity. From the moment of microfracture generation until the shear wave due to micro-rupture propagates to the inner boundary of the micro-rupture action region, denoted as t_s_^i^, the duration of the acoustic emission event t_d_^i^ is two times of t_s_^i^.

Recalculation of the moment tensor at each time step during the duration of the acoustic emission event t_d_^i^; If no new microfracture is produced in the t_d_^i^, the acoustic emission event contains only one microfracture; if a new microfracture is produced in the t_d_^i^ and its area of action overlaps with the old microfracture area of action, the microfracture is considered to belong to the same acoustic emission event and this acoustic emission contains multiple microfractures while the source particle area is superimposed and the duration is recalculated and extended. Eventually, the characteristics of time, spatial and rupture intensity of the acoustic emission events during the rupture of the specimen can be obtained by the above process.

## Evolution characteristics of coal body deformation and macro damage

**Figure 10:**
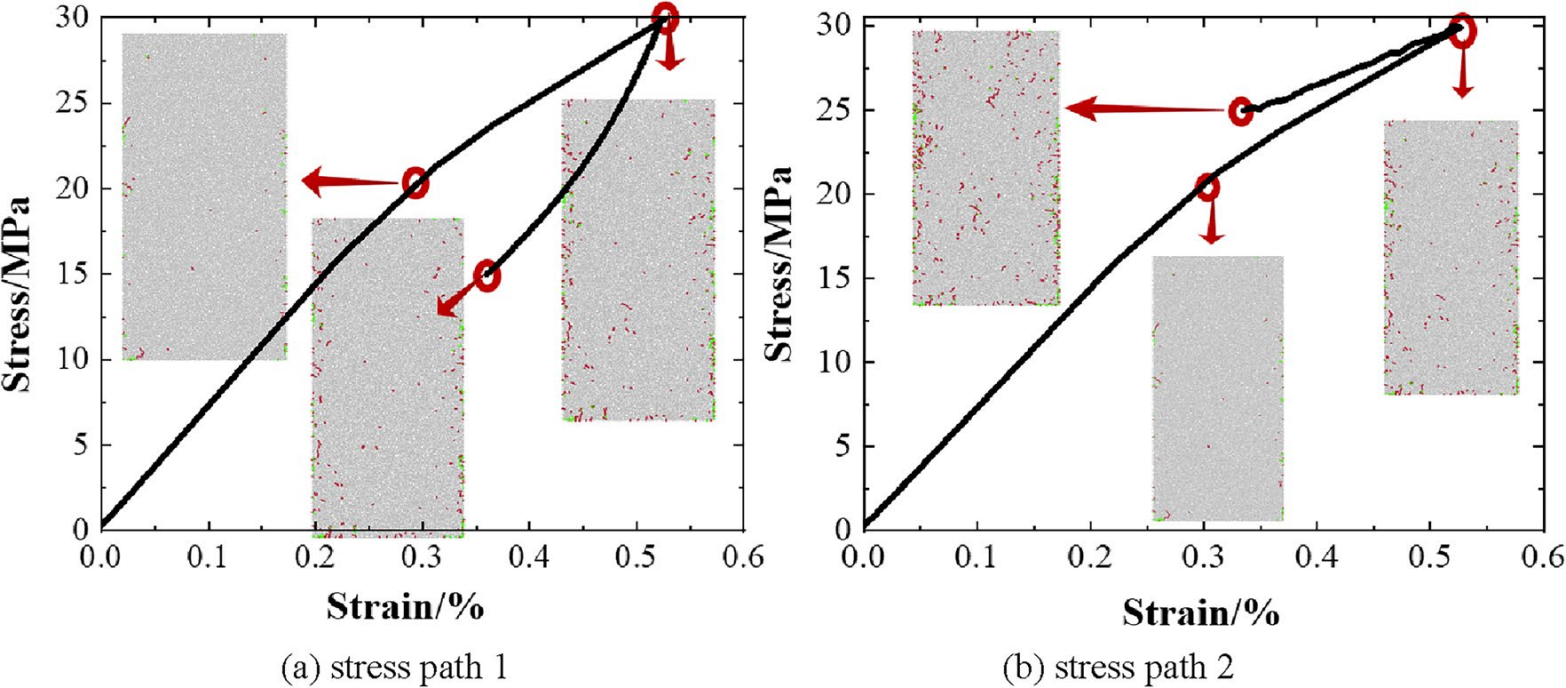
Stress–strain curve and crack evolution of specimen model.

As shown in Fig. 10, the stress–strain curves of the sample model under the action of stress path 1 and path 2 and the macro damage evolution characteristics at the end of different loading stages are shown respectively, according to the analysis:

1. The stress–strain curves of the sample models under the action of different stress paths show diametrically opposite characteristics at the post mining pressure relief stage, which indicates that the difference in deformation activation of the sample models under the action of the two stress paths is mainly reflected in the post mining pressure relief stage. Under the action of path 1, the axial strain of the sample model slowly decreases after entering the post mining pressure relief stage, the absolute value of the slope of the stress–strain curve at this stage is larger than that at the mining disturbance stage,this is because the sample model is unloaded simultaneously under axial pressure and confining pressure at this stage, so the compression deformation of the sample model is released. Under the action of path 2, the axial strain of the sample model after entering the post mining pressure relief stage decreases sharply, and the absolute value of the slope of its stress–strain curve is smaller than that in the mining disturbance stage, It indicates that the deformation of the sample model is rapidly decreasing, because the sample is in the stress state of axial pressure unloading and confining pressure loading at this stage, so the axial strain of the sample model is rapidly decreasing. The deformation of the sample model at the end of the post mining pressure relief stage of the two stress paths is 0.3793% and 0.3324%, respectively, it indicates that the pressure relief expansion of the sample model under the action of path 2 is restrained compared with path 1.
2. The in-situ stress recovery stage and the mining disturbance stage under the two stress paths show the same damage characteristics. At the in-situ stress recovery stage, fracture development appeares in the sample models, and the number of fractures is 63, most of them are distributed in the contact part between the sample model and the loaded wall, and a few cracks are scattered in the sample. In the mining disturbance stage, the cracks of the sample model grow rapidly, and the number of cracks increased from 63 to 323, besides the crack growth in the contact part between the sample model and the loaded wall, the crack distribution is obviously transferred to the inside and center of the sample model, and the internal damage of the sample model is obviously developed.
3. The difference of the damage characteristics of the sample models under the two stress paths is mainly reflected in the post mining pressure relief stage. Under the action of path 2, the damage development of the sample model increases significantly, and the fracture distribution further expands to the inside of the sample model on the basis of the mining disturbance stage, it shows that the damage development of the sample model is obvious at this stage. However, under the action of path 1, the damage development of the sample model is not obvious, which may be inconsistent with the above deformation law and the actual site, the reason caused the problem is that the contact mode between particles in the numerical simulation is a parallel bonding model. During the process of applying stress, the bonding between particles breaks and cracks are generated,when the stress is released in the post mining pressure relief stage, the destructive force of the inter particle bonding mode weakens, the damage will not continue to develop and the bonding between the damaged particles will not recover. In reality, there are two kinds of fractures in the coal body: primary fractures and secondary fractures, In the process of gradual reduction of axial pressure and confining pressure in the post mining pressure relief stage, some closed primary fractures of the coal body will be recovered, and some secondary fractures of the coal body under compression will also be recovered and increased in size. Therefore, due to the difference between the sample model and the internal structure of the coal, the damage development of the sample model in the action of path 1 is inconsistent with the deformation law and the actual site.

However, the fact that the damage of the sample model is not obvious in the post mining pressure relief stage of stress path 1 can indirectly indicate that the sample model is in an obvious pressure relief expansion state compared with the mining disturbance stage.

## Meso damage of coal body and evolution characteristics of gas migration channel

### Meso damage characteristics of coal under different stress paths

Under the same conditions, the permeability is mainly affected by the number and size of gas migration channels for coal body with different stress states, and the change of the number and size of gas migration channels in coal body is affected by the number and size of fractures and other damage states, therefore, the coal body is always in the coupled state of "stress field-fracture field-seepage field" under the mining disturbance. Therefore, on the basis of the above macro research, this chapter makes a more micro analysis from the distribution and types of damage (cracks), the location and relative energy of induced acoustic emission events when cracks occur, the size and direction of the microcrack force, the number of cracks and porosity of the sample model during the whole loading process to reflect the evolution characteristics of the gas migration channel in the sample model.

**Figure 11:**
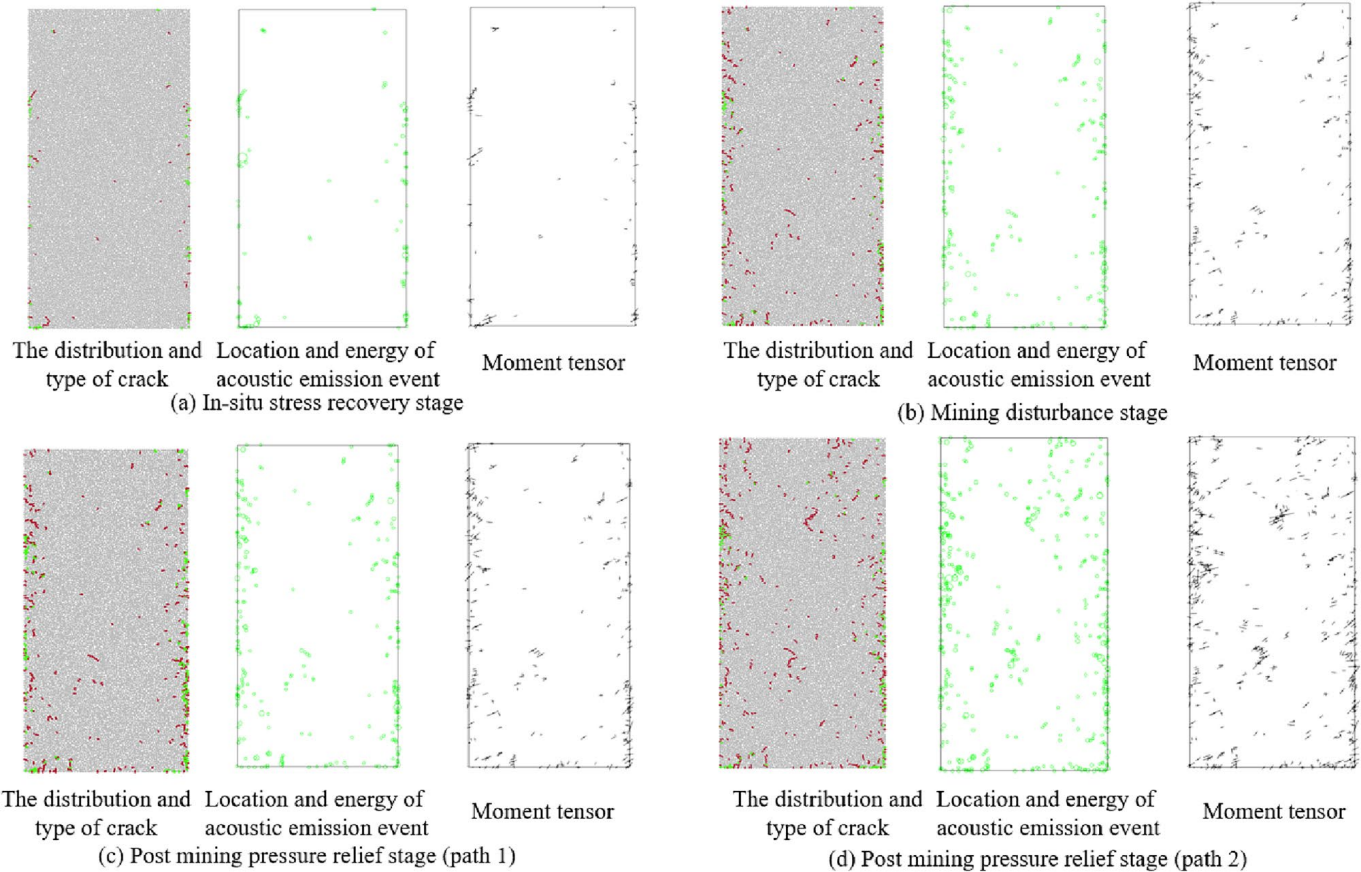
Evolution law of meso parameters in different stress stages.

The evolution laws of the meso damage parameters of the sample model at different stress stages under the action of stress paths 1 and 2 are shown in the Fig. 11, among the crack distribution and types, red and green dots represent the location of cracks, red represents shear cracks, and green represents tensile cracks, in acoustic emission events, a circle represents an acoustic emission event, and the size of the circle represents the energy of the acoustic emission event, the direction of the arrow in the moment tensor represents the direction of the force, and the length represents the magnitude of the force, Through the systematic analysis of Fig. 11, we can see that:

1. At the stage of in-situ stress recovery, tensile microcracks and shear microcracks coexist, and the number of tensile microcracks is more.The acoustic emission event location, energy and crack distribution, displacement show good correspondence, according to the energy magnitude and moment tensor of the micro fracture event, the energy of the micro fracture event in the in-situ stress recovery stage is small, and the micro fracture displacement is small.
2. In the mining disturbance stage, the crack distribution gradually develops toward the center of the sample model, it can be seen from the crack distribution and types that the shear crack growth is obvious in this stage, and the micro fracture displacement and acoustic emission event energy are increased compared with the previous stage.
3. As described above, the crack growth of the sample model is not obvious after it enters the post mining pressure relief stage under the action of the stress path 1 because of the internal structure of the sample model is different from that of the real coal body, however, through the analysis of the distribution and types of a small number of growing cracks, it can be seen that the tensile crack growth is obvious in this stage, which indirectly indicates that the sample model is in a pressure relief expansion state, and the displacement and acoustic emission event energy of the new crack are similar to those in the previous stage.

After the sample model enters the post mining pressure relief stage under the action of stress path 2, the number of cracks increases significantly, and according to the types of cracks, it can be seen that the increased cracks compared with the previous stage is mainly shear cracks, the displacement and acoustic emission event energy of the crack have increased compared with the previous stage, but they are not obvious, which indicates that the sample model is in the state of compression damage.

**Figure 12:**
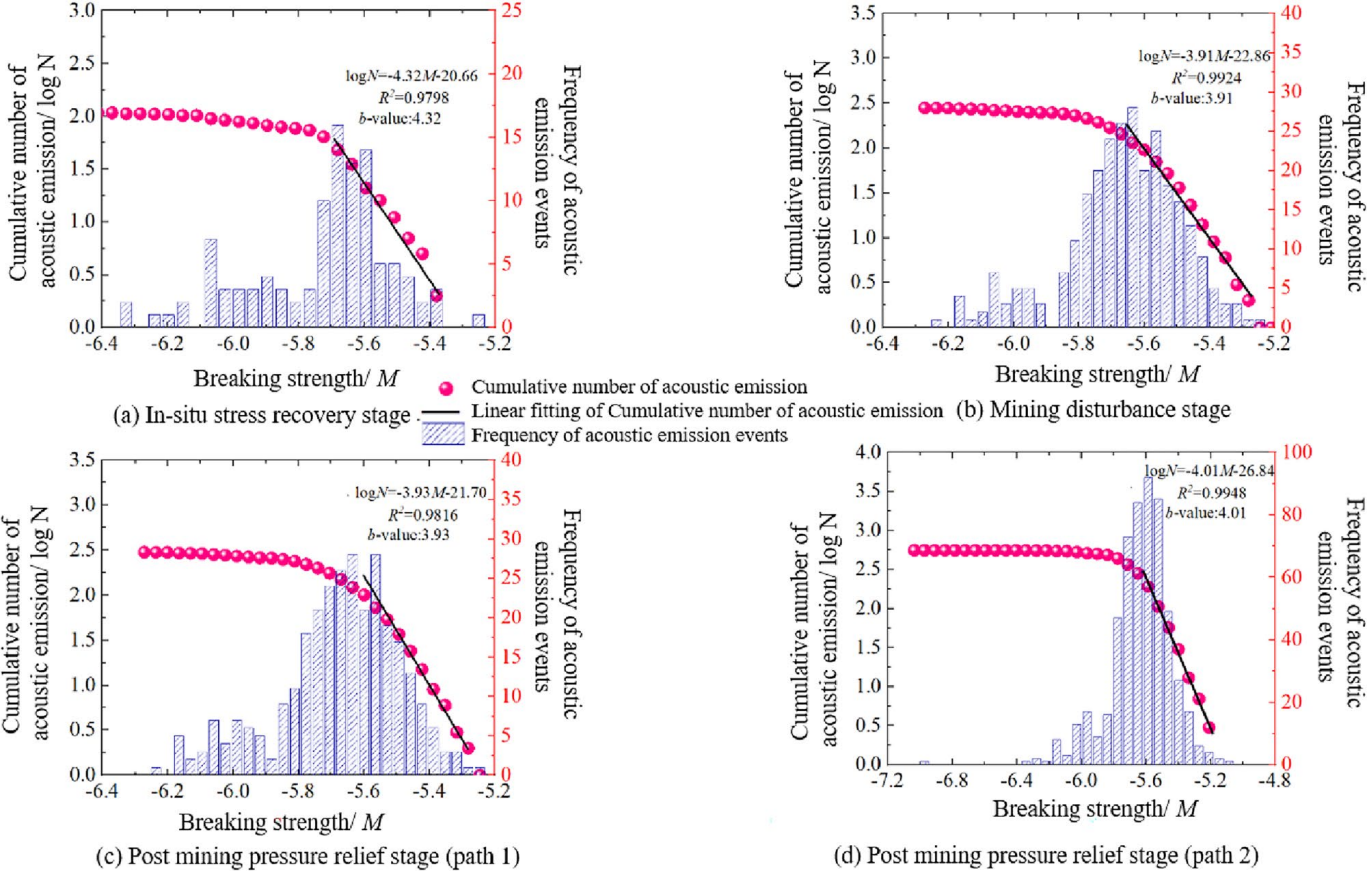
Relationship between the number of acoustic emission events and fracture strength of specimen model at different stress stages.

The relationship between the number of acoustic emission micro fractures and the fracture strength of the sample model at different stress stages of stress path 1 and stress path 2 is shown in Fig. 12, we can see that:

1. Under the action of two kind of stress paths, the number of acoustic emission events of the sample model at each stress stage is normally distributed with the breaking strength of the bond. Taking the in-situ stress recovery stage as an example, the maximum and minimum breaking strengths of acoustic emission events of the sample model are − 5.25 and − 6.25, when the breaking strength is − 5.70, the number of acoustic emission events is the largest, while when the breaking strength of acoustic emission events is the largest and the smallest, the number of acoustic emission events reaches the minimum. When the breaking strength of acoustic emission events is between − 5.25 and − 5.70, the number of acoustic emission event gradually increases with the decrease of the breaking strength, and the cumulative number of acoustic emission events presents a linear logarithmic relationship with the decrease of the breaking strength, in the C-M coordinate system, the slope of the approximate straight line of the acoustic emission accumulation number is about − 4.32, as shown in Fig. 11a. When m = − 5.70 ~ − 6.25, with the decrease of fracture strength, the number of acoustic emission decreases rapidly, and the cumulative number of acoustic emission approximates a straight line, and the slope gradually turns to 0.
2. The differences among the relationship of the number of acoustic emission events and the fracture strength of the sample model at the end of different stress stages are compared, mainly shown in: ① With the sequence of stress stages experienced by the sample model, the distribution range of acoustic emission event intensity gradually tends to be concentrated, that is, the range of fracture strength gradually decreases, it shows that during the process of the sample model going through the in-situ stress recovery stage, the mining disturbance stage and the post mining pressure relief stage, the strength of acoustic emission fracture event gradually tends to approach the strength corresponding to the most acoustic emission times; ② The breaking strength corresponding to the maximum number of acoustic emission and the slope of the approximate straight line of acoustic emission cumulant are gradually increasing, which indicates that the number of high-energy rupture events is gradually increasing.
3. The different relationship between the number of micro fractures and fracture strength under the two stress paths is also reflected in the post mining pressure relief stage, which is mainly shown as follows: The distribution range of acoustic emission event strength gradually tends to be more centralized under the action of stress path 2. The maximum and minimum breaking strengths of acoustic emission events of the sample model at the end of the post mining pressure relief stage are − 5.15 and − 6.35, although the distribution range is almost the same as that of the stress path 1 at the post mining pressure relief stage, but it is mainly concentrated between − 5.40 and − 5.80, it indicates that the acoustic emission fracture strength of the sample model tends to be more centralized.

### Evolution characteristics of gas migration channels under different stress paths

**Figure 13:**
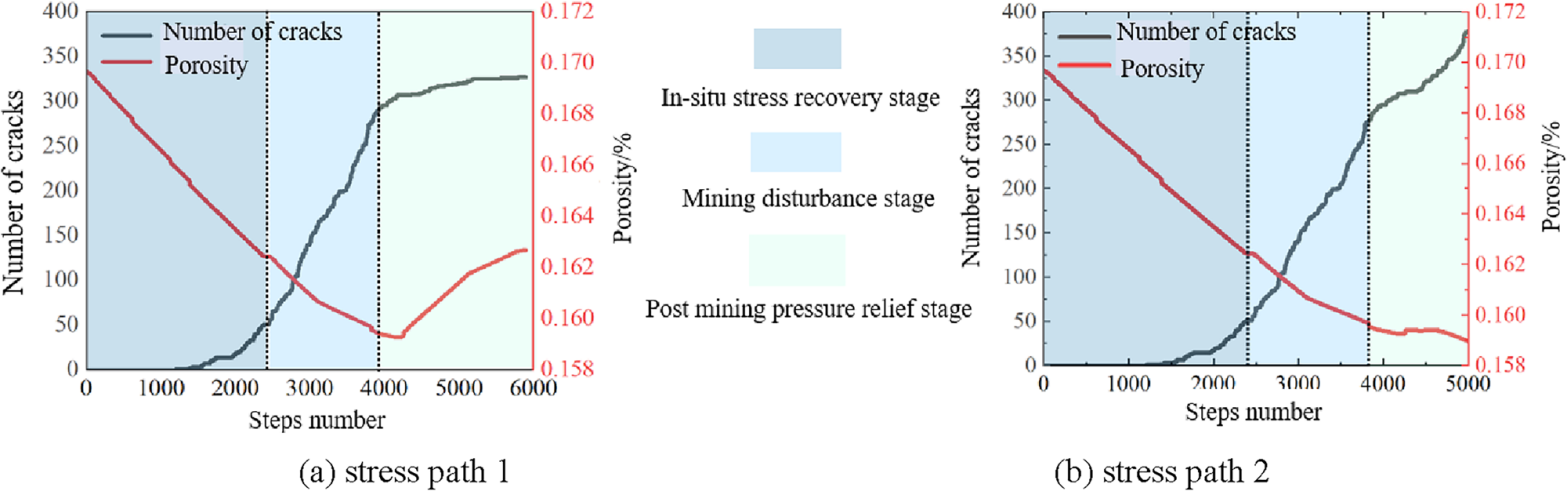
Evolution law of crack number and porosity at different stress stages.

As shown in Fig. 13, the number of cracks and porosity evolution laws of the sample model under the action of the two stress paths are as follows, which are used to reveal evolution characteristics of gas migration channels:

1. In the in-situ stress recovery stage, the porosity of the sample model gradually decreases due to the simultaneous loading of axial pressure and confining pressure, and the number of cracks starts to grow slowly in the middle and late parts of this stage. After entering the mining disturbance stage, the porosity of the sample model continues to decrease, while the number of cracks increases rapidly, which indicates that the sample model is in an obvious development state of compression damage in this stage, and the number of gas migration channels continues to decrease, which is consistent with the deformation and damage activation effects of the sample in the above study.
2. The difference of the crack number and porosity evolution law of the sample model under the two stress paths is also mainly reflected in the post mining pressure relief stage. The number of cracks in the sample model under the action of path 2 shows a trend of slow growth first and then rapid growth in the post mining pressure relief stage, and the porosity measured by the measurement circle shows a trend of slow growth first and then rapid reduction, which indicates that the sample model under the action of stress path 2 is in the compression damage stage in the post mining pressure relief stage, and the gas migration channel is also decreasing; After entering the post mining pressure relief stage under the action of stress path 1, although the number of cracks in the sample model increases unreasonably due to the influence of the internal structure of the sample model, the porosity measured by the measurement circle recovers significantly at this stage, indicating that the number of gas migration channels increases and the effect of pressure relief activation and antireflection is obvious, this verifies the conclusion that the above analysis that the damage in the post mining pressure relief stage is not obvious can indirectly indicate that the sample model is in the pressure relief expansion state compared with the mining disturbance stage.

## Conclusion

1. In the in-situ stress recovery stage and the mining disturbance stage of the two stress paths, the damage characteristics of the sample models are basically the same. The distribution of acoustic emission events concentrates in the high-intensity area and the porosity continues to decrease with the sequence of stress stages experienced by the sample model, the growth of the crack number in the in-situ stress recovery stage is slow, mostly tensile cracks, and the growth of the crack number in the mining disturbance stage is intensified, mostly shear cracks.
2. The deformation and macro damage characteristic of the sample models at the post mining pressure relief stage of the two stress paths are different. Compared with path 1, the pressure relief expansion effect of the sample model at this stage under the action of path 2 is suppressed, and the compression damage continues to develop.
3. The micro damage of the samples under the two stress paths at the post mining pressure relief stage is different, mainly shown as follows: at this stage of path 1, the crack number of sample model has little growth, and most of them are small energy tensile cracks, and the porosity increases, which verifies its obvious pressure relief activation effect; At this stage of path 2, the crack growth of the sample model is obvious, and most of them are high-energy shear cracks, and the porosity continues to decrease.

## Data Availability

The datasets used and/or analyzed during the current study available from the corresponding author on reasonable request.
